# Correlations between elevated basal sperm DNA fragmentation and the clinical outcomes in women undergoing IUI

**DOI:** 10.3389/fendo.2022.987812

**Published:** 2022-09-02

**Authors:** Chunhui Zhu, Shengmin Zhang, Fang Chen, Hong She, Yun Ju, Xidong Wen, Yurong Ji, Yu Pan, Chunxia Yang, Yan Sun, Naijun Dong, Kaifeng Liu, Feng Li, Tongmin Xue, Hengmi Cui

**Affiliations:** ^1^ Department of Reproductive Medicine Center, Northern Jiangsu People’s Hospital Affiliated to Yangzhou University/Clinical Medical College, Yangzhou University, Yangzhou, China; ^2^ Institute of Epigenetics and Epigenomics, College of Animal Science and Technology, Yangzhou University, Yangzhou, China

**Keywords:** AIH, IUI, sperm DNA fragmentation, SCSA, ROC curve

## Abstract

**Objective:**

This study aimed to explore the impact of the sperm DNA fragmentation index (DFI) on the clinical outcomes in women undergoing artificial insemination by husband intrauterine insemination (AIH-IUI).

**Methods:**

In this retrospective study, the value of sperm DFI was detected by sperm chromatin structure assay (SCSA) in a semen analysis collected before fertility treatment (basal DFI) in 1,500 IUI cycles at the infertility clinic of Northern Jiangsu People’s Hospital Reproductive Medicine Center from Jan 2016 to April 2021. Receiver operating characteristic (ROC) curves were used to calculate the cut-off value for the clinical outcomes of IUI, including the biochemical pregnancy rate, clinical pregnancy rate, delivery rate, and live birth rate, and multivariate logistic regression was conducted to analyse the risk factors for clinical outcomes after IUI.

**Result:**

In 1,500 IUI cycles, the results showed that there were no statistically significant differences between the normal DFI group and the abnormal DFI group in biochemical pregnancy rate (14.41% vs. 11.3%, *P* = 0.386), clinical pregnancy rate (12.9% vs. 10.5%, *P* = 0.433), delivery rate (11.0% vs. 8.9%, *P* = 0.456), live birth rate (10.9% vs. 8.9%, *P* = 0.484) or pregnancy loss rate (14.6% vs. 15.4%, *P* = 1.000).

**Conclusion:**

Sperm DFI alone may have limited predictive power for IUI clinical outcomes.

## Introduction

The process of spermatogenesis is complex, and multiple factors may lead to dysfunction of spermatogenesis, which ultimately leads to fertilization failure (1). Sperm DNA integrity is crucial for fertilization and the development of healthy offspring, and more and more reports emphasize the direct relationship between sperm DNA damage and male infertility (2). Sperm DFI can reflect the integrity of sperm DNA, and is an important indicator to assist in the evaluation of semen quality after the traditional semen analysis (3, 4). With the continuous development of science and technology, many new technologies have been applied to the examination of sperm DFI in clinical practice, including the TUNEL (terminal deoxynucleotidyl transferase dUTP nick end labeling) assay, Comet assay, SCSA (sperm chromatin structure assay), SCD (sperm chromatin dispersion) test, etc. (2, 5). Many studies show that high sperm DFI is associated with fertilization failure (6), delay embryonic development (7), lower high-quality blastocyst formation (8) and recurrent pregnancy loss (RPL) (9).

Infertility has become an important reproductive health problem in recent years, afflicting approximately 15% of couples at reproductive age worldwide (10). Infertility has been a neglected health issue for a long time partly because of the one-child policy in mainland China. A reproductive health survey found that the prevalence of infertility was approximately 15.5% (11). By the end of 2019, there were 517 assisted reproductive centers and 27 human sperm banks in mainland China. China’s ART cycles exceeded 1 million in 2016 (12) and reached 1.15 million in 2017 (13).

Artificial insemination (AI) refers to the technology of injecting the optimized sperm from the husband or the donor into the female reproductive tract so that the sperm and the egg are naturally combined to obtain pregnancy. With the development of artificial insemination as early as more than 200 years ago, human beings began to explore artificial insemination technology (14, 15). The first documented application of artificial insemination was presented in London in the 1770s by John Hunter (16). In 1954, Bunge and Sherman in the United States reported for the first time that frozen semen artificial insemination resulted in pregnancy, and the development of artificial insemination technology has also entered a new stage (17). The successful application of sperm freezing technology provides conditions for the preservation of male fertility and the storage and transportation of donated semen, and artificial insemination technology for sperm has been applied on a large scale. Since artificial insemination technology is closer to natural conception, it has the advantages of noninvasiveness, simplicity and convenience, making most patients more acceptable and more compliant, and it is also the preferred adjuvant treatment in clinical practice (18, 19). Intrauterine insemination (IUI) is safer and more cost-effective in clinical practice than other ARTs (20).

Although IUI has experienced a long time in clinical practice, the research on the influence of sperm on it is still ongoing. Many studies have found that the biochemical pregnancy rate, clinical pregnancy rate, and delivery rate of IUI in the high DFI group are lower than those of the normal sperm DFI group (21, 22), and other studies have found that the sperm DFI has no effect on IUI clinical pregnancy (18, 23). The effects of sperm DFI on the clinical outcome of IUI are still controversial. In this retrospective study, the sperm DNA fragmentation was detected in raw semen of men with IUI cycles to investigate the effect of sperm DNA fragmentation on the clinical outcome of IUI. This will provide a reference for the clinical application of sperm DFI in IUI.

## Materials and methods

### Study population

A total of 4,499 male semen samples were collected from January 2016 to April 2021 in the Reproductive Center of Subei People’s Hospital, and 1,500 cycles of clinical cases of couples were treated by IUI. Inclusion criteria: 1) Infertile couples completed all previous examinations, no abnormality in chromosomal examination, and the woman’s fallopian tubes were unobstructed (at least one side was unobstructed); 2) The female had dominant follicle development and ovulation; 3) Male sexual dysfunction, mild oligospermia and so on. Regarding grouping, men were divided into the normal sperm DFI group (DFI < 30%) and the abnormal sperm group (DFI ≥ 30%) according to their sperm DFI levels (5, 22). The clinical data of patients was collected, including age, body mass index (BMI), infertility duration, etc. All patients signed an informed consent form related to IUI and the studies involving human participants were reviewed and approved by hospital ethics committee (2021ky068).

IUI was performed in natural cycles if infertile women with regular menstruation and normal ovulation. For those with ovulation disorders, abnormal follicular development or prolonged menstrual cycle, after vaginal ultrasound examination on the third to fifth day of menstrual cycle, oral clomiphene (CC) or letrozole (LE) alone or in combination with gonadotropins or gonadotropins alone were used to stimulate ovaries to induce ovulation. From the 8th day of menstrual cycle, the growth of follicles was dynamically monitored under vaginal B-ultrasound. When follicles with a diameter of about 18mm appeared, 5,000-10,000 IU of human chorionic gonadotropin (hCG) was injected intramuscularly to induce ovulation, and IUI was carried out 36-42 hours after hCG injection.

### Collection semen and routine analysis

The men were abstinent for 2-7 days, and sperm were collected by masturbation. Routine semen processing analysis was performed according to the Laboratory Manual for Human Semen Examination and Processing, 5th Edition (24). The semen quality was analyzed and recorded by using a computer-aided semen analyzer (Beijing Suijia Software Co., Ltd).

### Analysis of sperm DNA fragmentation index

Sperm DNA fragmentation assay (SDFA) was performed using the sperm chromatin analysis (SCSA) kit (Zhejiang Cellpro Biotech Co., Ltd., Ningbo, China) in strict accordance with the product instructions (5, 25). The detailed analysis process was as follows. First, an appropriate volume of semen was added to 0.1 ml of solution A (TNE buffer, sperm dilution) and mixed. Then, 0.2 ml of solution B (acid solution of 0.1% Triton X-100, 0.15 mol/L NaCl, and 0.08 mol/L HCl, pH 1.2) was added and mixed. After standing for 30 s, 0.6 ml of acridine orange (AO) staining solution (6 μg/ml AO, 37 mmol/L citric acid, 126 mmol/L Na_2_HPO_4_, 1 mmol/L Na_2_EDTA, 0.15 mol/L NaCl, pH 6.0) was added and mixed. After the sperm were stained for 3 min, the sperm DFI was detected by a flow cytometer (FACS Calibur, BD Bioscience, San Jose, CA, USA). A minimum of 5,000 sperm were acquired, and the data were analyzed by using the software (DFIView 2010 Alpha11.15, CellPro Biotech, Ningbo, China). Total %DFI is Medium + High level of DNA fragmentation. The sperm DFI was expressed as the percentage of sperm with fragmented DNA compared to the total number of sperm. The variability of the replicate DFI measures was less than 5%.

### Semen optimization for IUI

The semen was collected 2 hours before IUI, the men abstained for 2-7 days, and semen was collected into a sterilized disposable wide-mouth collector. After checking each man’s name by fingerprint identification, the sperm spots were collected and placed in a 37°C incubator for incubation and liquefaction. After liquefaction, the semen was evaluated and recorded. The density gradient centrifugation method was used to optimize the semen. The specific operation steps were as follows: 1) The gradient centrifugation medium of 80% and 40% SpermGrad (Swedish Vitrolife Company) with two different concentrations was preheated in a 37°C incubator. 2) 1 ml of 80% high-concentration gradient centrifuge medium was added to the sterile conical centrifuge tube with a pipette, and then 1 ml of 40% low-concentration gradient medium was slowly added on top of it while being careful not to damage the interface between the two layers of gradient solution. Then, 2 ml of liquefied semen was added. According to the specific conditions of the semen, the amount of gradient centrifugation fluid was adjusted, or the number of centrifuge tubes was increased. 3) The samples were placed in a centrifuge at 300-400 × g for 15 minutes, the supernatant and gradient solution were removed, and only approximately 0.5 ml of sperm pellet was taken from the bottom. Then, 3 ml of upstream insemination solution was added, mixed well, and transferred to a Falcon 1006 centrifuge tube. 4) The tube was centrifuged at 300-400 × g for 5 minutes. The supernatant was removed, the sperm precipitate that was visible at the bottom of the tube was obtained, 0.5 ml of upstream insemination solution (IVF solution) was added, and the sperm suspension was evaluated and prepared for IUI use. The sperm DFI were tested before sperm preparation.

### Intrauterine insemination method

The patient was in the lithotomy position after emptying the bladder, washed the vulva with normal saline, and wiped the vagina, cervix, and fornix with cotton swab. A 1 ml syringe was connected with a disposable artificial insemination tube (COOK Company), 0.5 ml of sperm suspension was carefully and gently placed in the uterine cavity through the cervix and about 1cm above the uterine cavity, and then the artificial insemination tube was slowly removed after a short stay. After the operation, the patient was instructed to raise the hip at an angle of approximately 30 degrees until 15-30 minutes of observation in bed, and then leave if there was no special discomfort.

### Follow-up of pregnancy outcomes

A blood test was performed 14-16 days after IUI to measure the β-hCG level in peripheral blood to determine whether a biochemical pregnancy (more than 5.0 mIU/ml is diagnosed as biochemical pregnancy) was present. The intrauterine pregnancy sac was observed by vaginal ultrasonography between the fourth week and fifth week after IUI. Luteal support (progesterone 20 mg to be taken orally every day) was given in the stimulation cycles starts from 48 hours after IUI until clinical pregnancy if β-HCG positive. Pregnancy loss included miscarriage, ectopic pregnancy and stillbirth (26). Biochemical pregnancy rate = number of biochemical pregnancy cycles/artificial insemination cycles *100%; Clinical pregnancy rate = number of clinical pregnancy cycles/number of artificial insemination cycles *100%; Delivery rate = number of delivery cycles/number of artificial insemination cycles *100%; Live birth rate = number of live birth cycles/number of artificial insemination cycles *100%; Pregnancy loss rate = number of pregnancy loss cycles/number of clinical pregnancy cycles *100%.

### Statistical analysis

Statistical analysis was performed using IBM SPSS Statistics 22.0 version 25 (IBM Corp., Armonk, NY, USA). Categorical variables are presented as frequencies and percentages, whereas continuous variables are reported as the means ± standard deviations (SDs) or as the medians and interquartile ranges (IQRs, 25th-75th percentile). The normality of the distribution of the variables was determined using the Kolmogorov-Smirnov (K-S) test. Normally distributed data were expressed as the means and SDs, while the medians and IQRs were used for nonnormally distributed data. Groups were compared with student’s t-test or Mann–Whitney U-test as appropriate. Correlation analysis was performed by the Pearson method or Spearman method. The chi-square or Fisher’s exact test was used to compare categorical variables. The odds ratios and their 95% confidence intervals (ORs, 95% CIs) were calculated to show the associations between each predictor and the risks for clinical outcomes. The receiver operating characteristic curve (ROC) and the area under the curve (AUC) were calculated by MedCalc version 17 (MedCalc Software, Mariakerke, Belgium). The cut-off point of the ROC was also calculated to obtain the sensitivity and specificity of the model. All tests were two-tailed, *P* < 0.05 was considered statistically significant, and *P* < 0.01 was considered extremely significant.

## Results

A total of 4,499 semen samples were collected from outpatients, for a total of 1,500 IUI cycles, including 208 cycles of biochemical pregnancy (13.9%), 191 cycles of clinical pregnancy (12.7%), and 163 cycles of childbirth (10.9%). There were 161 cycles of live birth (10.7%, including 2 cycles of twin pregnancy, with a multiple birth rate of 1.2%) and 28 cycles of miscarriage (14.7%). The specific statistical analysis results are as follows.

### Correlation analysis between sperm DFI and semen routine parameters

A total of 4,499 male semen samples were collected from outpatient clinics, and sperm DFI was detected. The Kolmogorov-Smirnov (K-S) test results showed that the data did not obey a normal distribution (*P* < 0.001), except for the sperm specific movement parameter of beat-cross frequency (BCF) (**Supplementary Table 1**). The correlation analysis showed that the sperm DFI was positively correlated with sperm immotility percentage (r = 0.451, *P* < 0.001), male age (r = 0.140, *P* < 0.001), semen volume (r = 0.089, *P* < 0.001), abstinence days (r = 0.07, *P* < 0.001) and percentage of sperm DNA high stainability (HDS) (r = 0.171, *P* < 0.001), and negatively correlated with sperm concentration (r = -0.330, *P* < 0.001), sperm progressive motility percentage (r = -0.465, *P* < 0.001), sperm nonprogressive motility percentage (r= -0.08, *P* < 0.001), and sperm specific motility parameters (curvilinear velocity (VCL), straight-line velocity (VSL), average path velocity (VAP), mean angular displacement (MAD), straightness (STR), amplitude of lateral head displacement (ALH), linearity of movement (LIN), wobble (WOB) and beat cross frequency (BCF)) (all *Ps* < 0.001) (**Table 1**).

**Table 1:** Correlation between sperm DFI and semen conventional parameters.

Variable	ρ value	*P* value
Male age (years)	0.140	<0.001**
Semen volume (ml)	0.089	<0.001**
Abstinence days (days)	0.070	<0.001**
Sperm concentration (10^6^/ml)	-0.330	<0.001**
Sperm progressive motility (%)	-0.465	<0.001**
Sperm nonprogressive motility (%)	-0.08	<0.001**
Sperm immotility (%)	0.451	<0.001**
Sperm high DNA stainability (%)	0.171	<0.001**
VCL (μm/s)	-0.440	<0.001**
VSL (μm/s)	-0.429	<0.001**
VAP (μm/s)	-0.460	<0.001**
BCF (times/s)	-0.439	<0.001**
MAD (degree)	-0.446	<0.001**
STR	-0.448	<0.001**
LIN	-0.423	<0.001**
WOB	-0.475	<0.001**
ALH (μm)	-0.418	<0.001**

VCL, curvilinear velocity; VSL, straight-line velocity; VAP, average pathway velocity; BCF, beat cross frequency; MAD, mean angular displacement; STR, straightness (VSL/VAP); LIN, linearity of movement (VSL/VAP); WOB, wobble (VAP/VCL); ALH, amplitude of lateral head displacement. **P<0.01.

### Comparison of general data between the normal and abnormal sperm DFI groups in IUI cycles

A total of 1,500 IUI cycles were divided into the normal DFI group (DFI < 30%) and the abnormal sperm DFI group (DFI ≥ 30%) according to the diagnostic criteria of the sperm DFI; 1,376 cases were in the normal group (91.7%), and 124 cases (8.3%) were in the abnormal group. Statistical analysis results showed that there was no significant difference between the normal DFI group and the abnormal sperm DFI group in basic data such as infertility duration (2.1 vs. 3.0, *P* = 0.601), female age (28.0 vs. 29.0, *P* = 0.133), male BMI (24.8 vs. 24.2, *P* = 0.851), and sperm HDS percentage (6.2% vs. 5.9%, *P* = 0.755). There was a statistically significant difference in male age (30.0 vs. 30.5, *P* = 0.022) between the normal DFI group and the abnormal sperm DFI group (**Table 2**).

**Table 2:** Comparison of general data between the normal DFI group and abnormal DFI groups in IUI cycles.

Variable	Normal group (n=1376)	Abnormal group (n=124)	Z	*P* value
Sperm DFI (%)	13.7 (9.8-18.8)	35.9 (32.9-46.4)	-18.467	0.000**
Infertility duration (years)	2.1 (2.0-3.23)	3.0 (2.0-3.08)	-0.523	0.601
Male age (years)	30.0 (28.0-32.0)	30.5 (28.0-32.0)	-2.296	0.022*
Female age (years)	28.0 (27.0-31.0)	29.0 (27.0-31.0)	-1.503	0.133
Male BMI	24.8 (22.3-27.4)	24.2 (23.2-16.8)	-0.188	0.851
Sperm high DNA stainability(%)	6.2 (4.1-8.8)	5.9 (4.3-10.9)	-0.312	0.755

DFI, DNA fragmentation index; BMI, Body mass index. *P<0.05, **P<0.01.

### Comparison of clinical outcomes between the normal and abnormal sperm DFI groups in IUI cycles

Among the 1,500 IUI cycles, there were 1,376 cases of normal sperm DFI, 194 cases of biochemical pregnancy (14.1%), 178 cases of clinical pregnancy (12.9%), 152 cases of delivery (11.0%), 150 cases of live birth (10.9%), and 28 cases of pregnancy loss (15.7%, including 20 cases of miscarriage, 6 cases of ectopic pregnancy and 2 cases of stillbirth). There were 124 cases of abnormal sperm DFI, 14 cases of biochemical pregnancy (11.3%), 13 cases of clinical pregnancy (10.5%), 11 cases of delivery (8.9%), 11 cases of live birth (8.9%), and 2 cases of miscarriage (15.4%). The results showed that there were no statistically significant differences between the normal sperm DFI group and the abnormal sperm DFI group in the biochemical pregnancy rate (14.1% vs. 11.3%, *P* = 0.386), clinical pregnancy rate (12.9% vs. 10.5%, *P* = 0.433), delivery rate (11.0% vs. 8.9%, *P* = 0.456), live birth rate (10.9% vs. 8.9%, *P* = 0.484), or pregnancy loss rate (15.7% vs. 15.4%, *P* = 1.000) (**Table 3**). Further subgroup classification comparison (715 natural cycles and 785 stimulated cycles), there were no statistically significant differences in clinical outcomes between the normal and abnormal sperm DFI groups (**Supplementary Table 2**).

**Table 3:** Comparison of clinical outcomes between the normal and abnormal sperm DFI groups in IUI cycles.

Variable	Normal group(DFI<30%)	Abnormal group(DFI≥30%)	χ2	*P* value
Biochemical pregnancy	14.1% (194/1376)	11.3% (14/124)	0.751	0.386
Clinical pregnancy	12.9% (178/1376)	10.5% (13/124)	0.616	0.433
Delivery	11.0% (152/1376)	8.9% (11/124)	0.556	0.456
Live birth	10.9% (150/1376)	8.9% (11/124)	0.489	0.484
Pregnancy loss	15.7% (28/150) ^#^	15.4% (2/13)	0.000	1.000

#: Pregnancy loss including 10 cases of miscarriage, 6 cases of ectopic pregnancy and 2 cases of stillbirth.

### Prediction of sperm DFI on clinical outcomes after IUI

Receiver operating characteristic (ROC) curves were constructed to assess the effectiveness of sperm DFI in predicting the clinical outcomes of IUI. A clinically acceptable threshold was calculated when sensitivity plus specificity were maximum. The AUC of the sperm DFI for predicting biochemical pregnancy was 0.537 (95% CI: 0.551-0.562, *P* = 0.077). The cut-off value of the ROC was 16.75%, which had the best sensitivity of 69.23% and specificity of 40.63%. The AUCs of the sperm DFI for predicting clinical pregnancy, delivery and live birth were 0.531 (95% CI: 0.506-0.557, *P* = 0.148), 0.533 (95% CI: 0.507-0.559, *P* = 0.150), and 0.532 (95% CI: 0.507-0.558, *P* = 0.166), respectively (**Table 4**). The ROC curves showed that the sperm DFI was not valuable in predicting pregnancy for patients after IUI (all areas under the ROC curve of clinical outcomes below 54%). According to the results of the multivariable logistic regression analysis, the impact of sperm DFI (OR = 0.986, 95% CI 0.968-1.004, *P* = 0.118) on clinical outcomes of IUI was limited despite the removal of some confounding factors (**Supplementary Table 3**).

**Table 4:** ROC curve analysis of the sperm DFI for IUI pregnancy.

	Area underROC curve	SE	95% CI	Cut-off value(%)	Sensitivity(%)	Specificity(%)	*P* value
Biochemical pregnancy	0.537	0.021	0.551-0.562	16.75	69.23	40.63	0.077
Clinical pregnancy	0.531	0.022	0.506-0.557	15.42	62.30	46.45	0.148
Delivery	0.533	0.023	0.507-0.559	15.42	63.19	46.37	0.150
Live birth	0.532	0.023	0.507-0.558	15.42	62.73	46.30	0.166

ROC, receiver operating characteristic; CI, confidence interval.

## Discussion

With the improvement of people’s education level, changes in lifestyle, live environment, fertility concepts, and the aging of the social population, the number of births has declined in China. China proposed the implementation of the two-child fertility policy for couples where either the husband or the wife is from a single-child family in 2013, and a universal two-child policy was implemented in 2016 and then to the three-child policy proposed in 2021 (27, 28). It shows that the state hopes to promote the growth of the birth population. On the other hand, the increase in the number of assisted reproductive institutions approved by the state can alleviate the negative impact of ‘cannot birth’, but the solution to the problem of infertility still needs the advancement of assisted reproductive technology. The optimized sperm was sent into the woman’s uterine cavity through IUI to achieve the process of natural fertilization, pregnancy and childbirth, which is one of the commonly used assisted reproductive technologies, and the largest comprehensive analysis integrating success, risks and costs shows that IUI is safer and more cost-effective than other ART treatments (19, 20). IUI pregnancy rates have been reported to be mixed and varied widely, ranging from 8% to 22% (29). There are many factors affecting artificial insemination results, and most studies focus on patient age (30), ovarian function and egg quality (31, 32), infertility duration, ovulation induction (33, 34), endometrial thickness (35), and the number of inseminations. As women age, fertility declines significantly, and the proportion of early miscarriage and chromosomal abnormalities increases significantly (36, 37). Although there are existing methods such as sperm concentration, motility, and morphology to evaluate male fertility (38), these parameters are not standardized to a high degree and are subjective (39). There is controversy about the clinical significance of sperm DFI detection indicators for IUI (40).

In this study, the sperm DFI data of 4,499 sperm samples were tested for normality, showing a skewed distribution. Correlation analysis results showed that the sperm DFI was positively correlated with the man’s age, semen volume, abstinence days, and immotile sperm percentage; and negatively correlated with nonprogressive motility percentage, sperm concentration, sperm progressive motility percentage, and the specific motility parameters of sperm (VCL, VSL, VAP, BCF, ALH, MAD, LIN, STR and WOB). Sperm specific motility parameters are negatively correlated with sperm DFI, which is consistent with the results of Le et al. (41). There are significant correlations between routine semen parameters and sperm function parameters, which are both indicators of sperm quality, but the focus of detection was different (42). The percentage of sperm HDS is another index in the process of sperm DFI detection by SCSA method, which reflected the immaturity of the sperm nucleus and has been proposed to be due to a sub-optimal histone to protamine ratio that affects sperm nucleus compaction and therefore makes it susceptible to DNA damage (5). The sperm DFI was positively correlated sperm HDS percentage. These results suggest that the occurrence of human sperm functional defects is not a single reason and may be multifactorial. There is a significant correlation between sperm DFI and sperm motility, that is, the risk of abnormal sperm function is higher in low-quality sperm, which may have a common mechanism with the two abnormal phenotypes. Therefore, this study supports sperm DFI as a supplement to routine semen analysis. The positive correlation between sperm DFI and age is consistent with the results of Moskovtsev et al., who found that sperm DFI increased linearly with increasing male age (43–47). Fertility among older men is increasing worldwide, especially with the liberalization of China’s second-child birth policy, and a large number of couples over the age of 40 are trying to use assisted reproductive technology to achieve fertility. A comprehensive analysis of semen quality should be carried out to fully assess male fertility.

This study analyzed the association of sperm DFI with clinical outcomes in 1,500 IUI cycles. According to the diagnostic criteria of sperm DFI, they were divided into the abnormal sperm DFI group and the normal sperm DFI group, of which 124 cases were abnormal (8.3%) and 1,376 cases were normal (91.7%). Statistical analysis results showed that there was no statistically significant difference in basic data, such as infertility duration, female age, male BMI, and sperm HDS percentage between the two groups. There was a statistically significant difference in male age between the two groups, but the difference was not large. Statistical analysis and comparison showed that the normal sperm DFI group had a higher biochemical pregnancy rate, clinical pregnancy rate, delivery rate and live birth rate than the abnormal sperm DFI group, but there were no significant statistical differences. This result is consistent with Yang et al. (23) and different from Bungum et al. (22), which may be related to the population. With regard to the sperm DFI, we observed an optimum cut-off point of 16.75% for IUI biochemical pregnancy and 15.42% for clinical pregnancy, delivery and live birth, but they were not significant. Logistic regression analysis showed little prognostic value in predicting clinical outcomes after IUI. Therefore, the effect of sperm DFI on IUI clinical outcomes needs to be studied in larger samples.

Sperm DNA as a carrier of paternal genetic information, plays an important role in fertilization and embryonic development (48). Sperm DFI can reflect the integrity of sperm DNA and is an important indicator to assist in the evaluation of semen quality after the traditional semen analysis (3, 4). Oguz et al. compared the effects of two commonly used sperm preparation methods (swim-up and gradient technique) on sperm DFI through SCD method, and the result showed that gradient method has no statistically significant reduction in the DNA fragmented sperm rate after preparation as compared to basal rates (49). This study results showed that the elevated of basal sperm DFI had no significant impact on the clinical outcomes of IUI, which may be related to the reduction in the DNA fragmented sperm rate during sperm preparation, although there is no significant statistical difference before and after gradient centrifugation. The molecular mechanism of sperm DNA fragmentation and its impact on IUI need to be further studied.

## Data availability statement

The original contributions presented in the study are included in the article/**Supplementary Material**. Further inquiries can be directed to the corresponding authors.

## Ethics statement

The studies involving human participants were reviewed and approved by Northern Jiangsu People’s Hospital ethics committee (2021ky068). The patients/participants provided written informed consent to participate in this study, and human tissues were obtained with informed consent.

## Author contributions

HC, TX and FL conceived the idea. CZ and SZ wrote the manuscript. FC, HS, YJu and XW analyzed the data. CY, YS, YJi, YP, ND and KL edited and revised the manuscript. All authors listed have made a substantial, direct and intellectual contribution to the work, and approved it for publication.

## Funding

This study was funded by the Jiangsu postgraduate training innovation project (No. KYCX17_1888) and the National Natural Science Foundation of China (No. 81773013).

## Acknowledgments

We are indebted to all the research doctor coordinators for their invaluable contributions to patient recruitment and data collection.

## Conflict of interest

The authors declare that the research was conducted in the absence of any commercial or financial relationships that could be construed as a potential conflict of interest.

## Publisher’s note

All claims expressed in this article are solely those of the authors and do not necessarily represent those of their affiliated organizations, or those of the publisher, the editors and the reviewers. Any product that may be evaluated in this article, or claim that may be made by its manufacturer, is not guaranteed or endorsed by the publisher.
