# Supplementary material for: Correlations between elevated basal sperm DNA fragmentation and the clinical outcomes in women undergoing IUI
Source: Front Endocrinol (Lausanne). 2022 Sep 2;13:987812. doi: 10.3389/fendo.2022.987812 (PMC9478029; doi:10.3389/fendo.2022.987812)
Supplement: Supplementary file 1 [file DataSheet_1.docx]

| Supplementary table 1. Normality test of the basic data | | | | | |
| --- | --- | --- | --- | --- | --- |
|  | Median | Mean | Skewness | Kurtosis | K-S  *P* value |
| Male age (years) | 31.00 (27.00-33.00) | 30.69 ± 5.06 | 1.800 | 5.841 | <0.001** |
| Semen volume (ml) | 4.00 (2.90-5.40) | 4.26 ± 2.03 | 0.863 | 0.854 | <0.001** |
| Abstinence days | 4.00 (3.00-5.00) | 3.91±1.42 | 0.778 | 0.178 | <0.001** |
| Sperm concentration (10^6^/ml) | 62.78 (33.37-95.3) | 67.68 ± 44.65 | 1.207 | 4.893 | <0.001** |
| Sperm progressive motility (%) | 46.00 (32.00-59.00) | 44.56 ± 18.57 | -0.232 | -0.557 | <0.001** |
| Sperm nonprogressive motility (%) | 6.00 (3.56-13.00) | 8.55± 6.71 | 1.233 | 1.652 | <0.001** |
| Sperm immotility (%) | 45.08 (32.00-60.49) | 46.90 ± 20.04 | 0.347 | -0.404 | <0.001** |
| DFI (%) | 15.87(10.19-24.87) | 19.39 ± 13.13 | 1.644 | 3.738 | <0.001** |
| HDS (%) | 6.08 (4.19-8.83) | 7.24 ± 4.83 | 2.740 | 12.838 | <0.001** |
| VCL (μm/s) | 32.77 (21.78-43.62) | 33.08 ± 15.28 | 0.193 | -0.256 | <0.001** |
| VSL (μm/s) | 16.05 (10.67-22.18) | 16.61 ± 8.31 | 0.362 | -0.162 | <0.001** |
| VAP (μm/s) | 22.88 (15.33-30.75) | 23.23 ± 10.72 | 0.127 | -0.446 | <0.001** |
| BCF (times/s) | 7.57 (5.35-9.83) | 7.58 ± 3.26 | 0.034 | -0.304 | 0.122 |
| ALH (μm) | 3.52 (2.49-4.55) | 3.61 ± 1.66 | 0.638 | 1.131 | <0.001** |
| MAD (degree) | 24.46(16.90-31.58) | 24.30±10.36 | -0.018 | -0.429 | <0.001** |
| STR | 0.40 (0.29-0.50) | 0.39±0.15 | -0.250 | -0.337 | <0.001** |
| LIN | 0.29(0.21-0.37) | 0.29±0.12 | 0.073 | -0.215 | <0.001** |
| WOB | 0.43(0.31-0.53) | 0.42±0.16 | -0.313 | -0.326 | <0.001** |
| Abbreviations: DFI, DNA fragmentation index; HDS, high DNA stainability; VCL, curvilinear velocity; VSL, straight-line velocity; VAP, average pathway velocity; BCF, beat cross frequency; MAD, mean angular displacement; STR, straightness (VSL/VAP); LIN, linearity of movement (VSL/VAP); WOB, wobble (VAP/VCL); ALH, amplitude of lateral head displacement. Note: ***P* < 0.01. | | | | | |

| Supplementary table 2. Comparison of clinical outcomes between the normal and abnormal sperm DFI groups in natural and stimulated IUI | | | | |
| --- | --- | --- | --- | --- |
| Variable | Normal group  （DFI＜30%） | Abnormal group（DFI≥30%） | χ2 | *P* value |
| **Type of cycles** |  |  |  |  |
| Natural cycle | 48.1% (662/1376) | 42.7% (53/124) | 1.314 | 0.252 |
| Stimulated cycle | 51.9% (714/1376) | 57.3% (71/124) |  |  |
| **Natural cycles** |  |  |  |  |
| Biochemical pregnancy | 12.1% (80/662) | 11.3% (6/53) | 0.027 | 0.869 |
| Clinical pregnancy | 10.6% (70/662) | 7.9% (6/53) | 0.029 | 0.865 |
| Delivery | 8.9% (59/662) | 7.5% (4/53) | 0.007 | 0.932 |
| Live birth | 8.9% (59/662) | 7.5% (4/53) | 0.007 | 0.932 |
| Pregnancy loss | 15.7% (11/70)^#1^ | 33.3% (2/6) | 0.286 | 0.593 |
| Miscarriage | 14.3% (10/70) | 33.3% (2/6) | 0.416 | 0.519 |
| **Stimulated cycles** |  |  |  |  |
| Biochemical pregnancy | 16.0% (114/714) | 11.3% (8/71) | 1.086 | 0.297 |
| Clinical pregnancy | 15.1% (108/714) | 9.9% (7/71) | 1.433 | 0.231 |
| Delivery | 13.0% (93/714) | 9.9% (7/71) | 0.582 | 0.445 |
| Live birth | 12.4% (91/714) | 9.9% (7/71) | 0.492 | 0.483 |
| Pregnancy loss | 15.7% (17/91) ^#2^ | 0.0% (0/7) | 0.345 | 0.557 |
| Miscarriage | 9.3% (10/98) | 0.0% (0/7) | 0.023 | 0.880 |
| #1: Pregnancy loss including a case of ectopic pregnancy and 10 cases of miscarriage; #2: Pregnancy loss including 10 cases of miscarriage, 5 cases of ectopic pregnancy and 2 cases of stillbirth. | | | | |

| Supplementary table 3. Multivariate logistic regression analysis of influencing factors for IUI clinical pregnancy. | | | | | |
| --- | --- | --- | --- | --- | --- |
|  | β | Wald | OR | 95% CI | *P* value |
| Sperm DFI (%) | -0.014 | 2.446 | 0.986 | 0.968-1.004 | 0.118 |
| Male age (years) | -0.002 | 0.006 | 0.998 | 0.937-1.063 | 0.940 |
| Female age (years) | -0.012 | 0.108 | 0.988 | 0.921-1.060 | 0.743 |
| Male BMI | 0.042 | 4.607 | 1.043 | 1.004-1.084 | 0.032* |
| Infertility years (years) | -0.117 | 4.932 | 0.890 | 0.802-986 | 0.026* |
| Sperm high DNA staining (%) | -0.007 | 0.200 | 0.993 | 0.961-1.025 | 0.654 |
| β: regression coefficient; OR: odd ratio; CI: confidence interval; ROC: receiver operating characteristic. Note: **P* < 0.05 | | | | | |
